# Detection of *EML4-ALK* in Lung Adenocarcinoma Using Pleural Effusion with FISH, IHC, and RT-PCR Methods

**DOI:** 10.1371/journal.pone.0117032

**Published:** 2015-03-18

**Authors:** Leilei Liu, Ping Zhan, Xiaodie Zhou, Yong Song, Xiaojun Zhou, Like Yu, Jiandong Wang

**Affiliations:** 1 Department of Pathology, Jinling Hospital, Nanjing University School of Medicine, Nanjing, China; 2 Department of Medicine, Nanjing Thoracic Hospital, Nanjing, China; 3 Department of Respiratory Medicine, Jinling Hospital, Nanjing University School of Medicine, Nanjing, China; Texas Tech University Health Sciences Center, UNITED STATES

## Abstract

Anaplastic lymphoma kinase (*ALK*) and echinoderm microtubule-associated protein-like 4 (*EML4*) gene rearrangements occur in approximately 5% of non-small-cell lung cancers (NSCLC), leading to the overexpression of anaplastic lymphoma kinase and predicting a response to the targeted inhibitor, crizotinib. Malignant pleural effusion occurs in most patients with advanced lung cancer, especially adenocarcinoma, and tissue samples are not always available from these patients. We attempted to clarify the feasibility of detecting the *EML4-ALK* fusion gene in pleural effusion cells using different methods. We obtained 66 samples of pleural effusion from NSCLC patients. The pleural effusion fluid was centrifuged, and the cellular components obtained were formalin fixed and paraffin embedded. The *EML4-ALK* fusion gene status was determined with fluorescent in situ hybridization (FISH), reverse transcription—polymerase chain reaction (RT-PCR), and immunohistochemistry (IHC). *EML4-ALK* was detected in three of 66 patient samples (4.5%) with RT-PCR. When the RT-PCR data were used as the standard, one false positive and one false negative samples were identified with IHC; and one false negative sample was identified with FISH. These results suggest that a block of pleural effusion cells can be used to detect the *EML4-ALK* fusion gene. IHC had good sensitivity, but low specificity. FISH had low sensitivity, but high specificity. RT-PCR is a good candidate method for detecting *EML4-ALK* in blocks of pleural effusion cells from lung cancer patients.

## Introduction

Lung cancer is the leading cause of cancer death worldwide. Its morbidity and mortality are increasing in both developed and developing countries [1,2]. Traditional therapies and improved diagnosis have not significantly increased overall survival. The discovery of specific molecular changes in certain lung cancers provides excellent opportunities for the development of new targeted therapies. The rearrangement of the anaplastic lymphoma kinase (*ALK*) gene and echinoderm microtubule-associated protein-like 4 (*EML4*) gene occurs in approximately 5% of lung adenocarcinomas, leading to the overexpression of anaplastic lymphoma kinase [3–5]. Novel tyrosine kinase inhibitors targeting ALK activity have proven anticancer activity, and crizotinib produces a good clinical response in advanced NSCLC patients carrying *ALK* rearrangements [6–9].

Several methods have been developed to detect *ALK* rearrangements, including fluorescent *in situ* hybridization (FISH), reverse transcription—PCR (RT-PCR), and immunohistochemistry (IHC). The Vysis LSI ALK Break Apart FISH Probe Kit (Abbott Molecular, Des, Plaines, IL) has become a Food and Drug Administration (FDA)-approved companion diagnostic agent to the ALK inhibitor, crizotinib, in a targeted therapy for lung cancers. However, FISH can be expensive and time-consuming and requires specialized fluorescence microscopy equipment and expertise. The *ALK* FISH assay, in particular, can be difficult to interpret because the most common alteration, an intrachromosomal inversion, leads to a subtle separation of the 5′ and 3′ signals. Moreover, cells with no rearrangement can display, not uncommonly, some nonspecific signal separation. As a result, the assay is prone to false negative and false positive results and shows significant interobserver variability. A multiplex RT-PCR system was developed to capture *ALK* fusion transcripts, and has been confirmed to be a reliable technique for the diagnosis of *EML4-ALK* [10]. *EML4-ALK* fusions drive the upregulation of anaplastic lymphoma kinase transcription and protein expression. Immunohistochemical detection of the ALK protein has been available for many years for the diagnosis of anaplastic large-cell lymphoma [11,12], but the antibodies traditionally used in the diagnosis of this lymphoma are insufficiently sensitive for detection at the level at which ALK is expressed in *ALK*-rearranged lung cancers. However, several recent studies have demonstrated that a relatively new anti-ALK clone, D5F3, can identify ALK-rearranged lung adenocarcinoma more accurately than FISH [13]. According to these studies, ALK expression can vary. Although strong staining with FISH seems to be 100% specific for the presence of the rearrangement, weak-to-intermediate staining has been reported in FISH-negative tumors. The basis for the discrepancies between *ALK* FISH and IHC is unclear[14]. Malignant pleural effusion occurs in most patients with advanced lung cancer, especially adenocarcinoma, but tissue samples are not always available from these patients. In this study, we collected 66 cell block samples of pleural effusion fluid from NSCLC patients and clarified the feasibility of detecting the *EML4-ALK* fusion in cell blocks from pleural effusion fluid with different techniques.

## Materials and Methods

### Patients and samples

Patients who were diagnosed cytologically as suffering NSCLC, and whose cell blocks were subjected to *EML4-ALK* rearrangement analysis in Jinling Hospital, China, between January 2013 and May 2014, were included in the study. In total, 66 consecutive cases of NSCLC were retrieved from the Department of Pathology. The clinicopathological characteristics of the patients were obtained from their medical records. The male/female ratio of the patients was 40/26. The median age of the patients was 56.5 years (range, 34–79 years). The study protocol was approved by the Jinling Hospital Clinical Research Ethics Committee, China. All of the patients signed a written informed consent form.

#### FISH

In current clinical trials of the ALK kinase inhibitor crizotinib, the break-apart FISH assay was used as the standard diagnostic test for detecting an *ALK* rearrangement. This test has been approved by the FDA and is the current gold standard technique. Formalin-fixed, 4 μm thick, paraffin-embedded cell blocks were used to evaluate the *ALK* genetic status with FISH. The slides were deparaffinized, dehydrated, immersed in 0.2 N HCl for 20 min, then washed and incubated in pretreatment solution at 80°C. In this assay, the 5′ and 3′ ends of the *ALK* gene are differentially labeled with red and green fluorescent probes, respectively. Under normal circumstances, the two probes are close together. *ALK* rearrangement is inferred if the orange and green signals are not touching (split). Splits that are less than two signal distances apart are classified as “rearranged”, given the nature of the 2p23 inversion. FISH analysis of *ALK* rearrangements was performed with the Break Apart FISH Probe Kit (China Medical Technologies, Inc., Beijing, China), according to the manufacturer’s instructions. Three viewers independently scored 100 nuclei on each slide to determine the interobserver agreement. The reference range for the *ALK* rearrangement was used in accordance with published papers. A cutoff of 15% positive cells was used to classify the samples as positive or negative for the *ALK* rearrangement.

#### IHC

The immunohistochemical detection of ALK has improved with the incorporation of techniques to enhance the immunohistochemical signal and the development of more-sensitive anti-ALK antibodies. IHC for ALK was performed on 4 μm thick formalin-fixed, paraffin-embedded sections of cell blocks of pleural effusion fluid. The cells were immunohistochemically stained according to the protocol provided by Ventana Medical Systems, Inc. and Roche Diagnostics International, Inc (1910 Innovation Park, AZ 85755, USA). Briefly, one serial tissue section was stained with hematoxylin and eosin (H&E), a second serial tissue section was stained with the Ventana anti-ALK (D5F3) antibody, and a third serial tissue section was stained with a negative control anti-rabbit Ig monoclonal antibody. The assay was performed with the OptiView DAB IHC Detection Kit and the OptiView Amplification Kit, and was run on the BenchMark ULTRA platform (1910 Innovation Park, AZ 85755, USA). Ventana ALK 2 in 1 Control Slides were used as the system-level control. The stained slides were reviewed by two pathologists. Staining was graded semiquantitatively, as follows: 0 for absent or barely perceptible expression; 1 (low) for weak to moderate multifocal expression; and 2 (high) for strong staining in most cells. All positive samples demonstrated a granular, cytoplasmic expression pattern. Focal, weak rimming of intracellular mucin droplets was considered negative.

#### EML4-ALK RT-PCR

Total RNA was extracted from the formalin-fixed, paraffin-embedded cell blocks with the RNeasy FFPE Kit (Qiagen, Hamburg, Germany). Reverse transcription and the detection of *EML4-ALK* was performed with the EML4-ALK Fusion Gene Detection Kit (AmoyDx, Xiamen, China). The primer sets used to amplify *EML4-ALK* variant 1, 3a/b, 2, 5′, and 5a/b are included in the kit. PCR was performed with the following parameters: denaturation at 95°C for 5 min; 15 cycles of 95°C for 25 s, 64°C for 20 s, and 72°C for 20 s; and 31 cycles of 93°C for 25 s, 60°C for 35 s, and 72°C for 20 s.

## Results

### ALK protein detected with Ventana IHC

**Fig 1:**
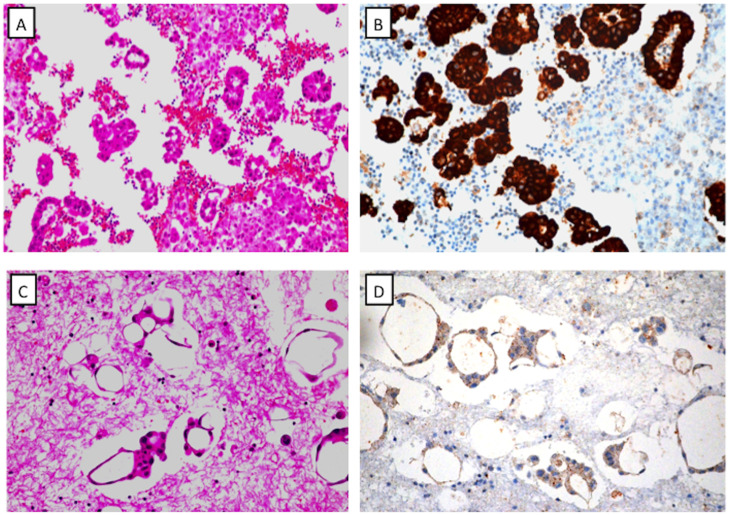
Representative examples of Ventana immunohistochemical staining of ALK. A: H&E staining of a cell block shows lung carcinoma cells. B: Strong staining of ALK protein in a cell block slide (corresponding to sample A). C: H&E staining of a cell block shows lung carcinoma. D: Weak staining of ALK protein in a cell block (corresponding to sample C).

ALK IHC was performed with the D5F3 antibody and was independently scored by two reviewers. Cell block slides of pleural effusion fluid were successfully stained with IHC to detect the ALK protein. ALK expression of score 0 was found in 53 samples, score 1 in 10 samples, and score 2 in three samples (Fig. 1).

### *EML4-ALK* rearrangement determined with FISH

**Fig 2:**
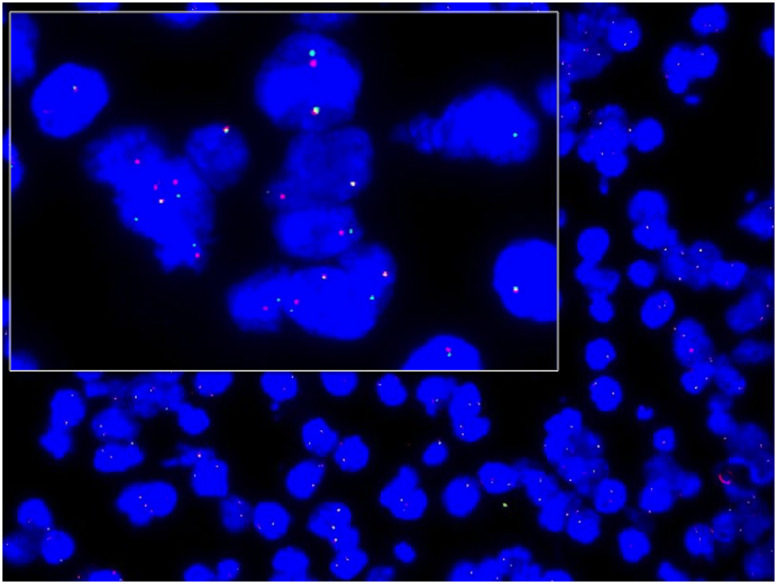
Detection of the *EML4-ALK* gene rearrangement with FISH. Dual-color, break-apart fluorescent *in situ* hybridization was performed on cell block slides. The centromeric (green) and telomeric (red) flanks of the *ALK* locus. Splitting the red and green signals indicates *ALK* rearrangement.

All cell blocks were analyzed with *ALK* FISH using break-apart probes targeting *ALK* at the 2p23 locus. The break-apart signal pattern, in which one fusion signal (orange) and single red and green signals are observed in the nucleus (> 15%), is considered positive in the FISH assay (Fig. 2). A few nuclei showing a predominant signal pattern indicating the deletion of the 5′ region (nuclei with a single red signal in addition to the fused signal) was also considered a positive FISH result. Fifty-three samples that stained negatively for ALK by IHC were negative on FISH; 10 samples with intermediate staining for ALK protein (IHC score 1) were negative on FISH; and two of the three samples with strong staining for ALK (score 2) were positive for *ALK* on FISH.

### *EML4-ALK* rearrangement detected with RT-PCR

We investigated the presence of the *EML4-ALK* fusion variant in 66 samples of cell blocks from pleural effusion fluid using RT-PCR, and identified 3 of the rearrangement-positive samples. The fusions were confirmed with direct sequencing. Among the three RT-PCR-positive samples, two were positive (score 2) according to both IHC and FISH, and one was positive (score 1) according to IHC.

### Specificity and sensitivity of IHC and FISH, against the RT-PCR standard

The *EML4-ALK* rearrangement was detected in 66 samples of cell blocks from pleural effusion fluid using Ventana IHC, RT-PCR, and FISH (Table 1). If the RT-PCR results were considered the gold standard for detecting the *EML4-ALK* gene rearrangement and the cut-off for ALK protein expression was an IHC score of 2, then the sensitivity and specificity of IHC were 75% and 98.4%, respectively. If the cut-off for ALK protein expression was an IHC score of 1, then its sensitivity was 100% and its specificity was 86.3%. The sensitivity and specificity of FISH were 75% and 100%, respectively.

**Table 1:** Detection of EML4-ALK in NSCLC by IHC, RT-PCR and FISH.

Ventana IHC	No.	RT-PCR		FISH test	
		+	-	+	-
**Score 0**	**53**	**0**	**53**	**0**	**53**
**Score 1**	**10**	**1**	**9**	**0**	**10**
**Score 2**	**3**	**2**	**1**	**2**	**1**

## Discussion

The therapeutic approach to lung cancer has changed dramatically in recent years with the widespread use of molecular testing, optimized to identify molecular targets for biological drugs [15–18]. Approximately 70% of patients with NSCLC are in the late stage of the disease, and for these patients, the only pathological material available for molecular testing may be cytological specimens [19–22]. Pleural effusion is observed in 7%–15% of lung cancer patients, and can be obtained noninvasively and more often than samples of the primary lesion. Although pleural effusion is a potential candidate for molecular testing, its use in detecting *EML4-ALK* rearrangements has not been well investigated [23–26].

Currently, FISH is considered the gold standard for screening NSCLC specimens for *ALK* rearrangements. However, according to FDA guidelines, this analysis is only applicable to histological samples. The Vysis LSI ALK Dual Colour, Break Apart Rearrangement Probe (Abbott Molecular, Abbott Park, IL) was the methodology used in the trials that led to the approval of crizotinib. Thus, the *ALK* fluorescent *in situ* hybridization test became the standard procedure for patient identification. However, FISH testing is generally acknowledged as relatively costly and time-consuming, and requires specialized equipment and considerable expertise [13,27]. Inversions in 2p lead to a relatively small physical separation of the two *ALK* fragments, which sometimes leads to false negative results. Fifty assessable cells must be read in *ALK* FISH, and at least 15% of them must show the rearrangement for a “positive test”. However, as many as 20% of routine diagnostic lung cancer samples that require testing fall short of this requirement [28].

RT-PCR is a highly sensitive and specific technique. In another study, the detection of the *EML4-ALK* fusion transcript by RT-PCR in a cell block identified a false negative *ALK* FISH result in the same cells [29]. The specific *EML4-ALK* fusion transcript detected was variant 1 (*EML4* exon 13 to *ALK* exon 20), which is the most common fusion in NSCLC. Consistent with these findings, others have reported difficulties in detecting *ALK* rearrangements with FISH in patients with the variant 1 fusion because the signal splits on FISH are small [30,31].

Immunohistochemistry is a potentially useful technique for prescreening patients for *ALK* FISH testing, reducing the burden of FISH testing to a manageable number. When high-affinity antibodies are used with a highly sensitive detection method, ALK IHC can provide an effective prescreening technique to complement *ALK* FISH testing. Different anti-ALK antibodies target ALK, including ALK1, 5A4, and D5F3 clones. ALK IHC is a valuable and cost-effective screening method that can help to initially identify patients with ALK rearrangements. However, it is important to appreciate the limitations of IHC, which include staining heterogeneity, variable or subtle cytoplasmic expression, and the need for highly specific protocols for lung tissues [29,32,33].

In NSCLC, the frequency of the EML4-ALK fusion gene is reported 5–7%[34–36]. Savic et al reported they detected ALK protein in 37.5% of cytological specimens with ALK immunocytochemistry[37]. In this study, the incidence of ALK rearrangements in pleural effusion cells of lung adenocarcinoma was low (4.5%) according to the RT-PCR results. This can be partially interpreted by that we used cell block samples were different from the tissue samples. Because of small cohort used, our data would be validated by further investigation.

RT-PCR is suited to detect EML4-ALK fusion transcripts, but is not available in many routine pathology departments in China. In addition, the cell block is stored as FFPE, in which case RNA may be substantially degraded. It is still possible that our multiplex RT-PCR analysis failed to efficiently amplify the longer product. Furthermore, RT-PCR may not detect all the translocations involving the gene, and that there may be as-yet-undetected fusion events for EML4-ALK in our samples.

The important goal of this study was to compare the sensitivity and specificity of three detection methods: IHC, FISH, and RT-PCR. It revealed that FISH has low sensitivity (75%), but very good specificity (100%). The sensitivity and specificity of IHC staining depend on the cut-off value selected. In general, IHC has better sensitivity and lower specificity than FISH. RT-PCR is the most sensitive method and can identify different variants or rearrangement partners.
