# Correction: Detection of *EML4-ALK* in Lung Adenocarcinoma Using Pleural Effusion with FISH, IHC, and RT-PCR Methods

**DOI:** 10.1371/journal.pone.0341293

**Published:** 2026-01-21

**Authors:** The PLOS One Editors

## Notice of Republication

This article [1] was republished on October 28, 2025, to address an issue identified post-publication.

Please download this article again to view the correct version.
